# Supplementary material for: Conditioning sharpens the spatial representation of rewarded stimuli in mouse primary visual cortex
Source: eLife. 2018 Sep 17;7:e37683. doi: 10.7554/eLife.37683 (PMC6141231; doi:10.7554/eLife.37683)
Supplement: Transparent reporting form [file elife-37683-transrepform.docx]

***eLife’s* transparent reporting form**

We encourage authors to provide detailed information *within their submission* to facilitate the interpretation and replication of experiments. Authors can upload supporting documentation to indicate the use of appropriate reporting guidelines for health-related research (see [EQUATOR Network](http://www.equator-network.org/%20)), life science research (see the [BioSharing Information Resource](https://biosharing.org/)), or the [ARRIVE guidelines](http://www.plosbiology.org/article/info:doi/10.1371/journal.pbio.1000412) for reporting work involving animal research. Where applicable, authors should refer to any relevant reporting standards documents in this form.

If you have any questions, please consult our Journal Policies and/or contact us: [editorial@elifesciences.org](mailto:editorial@elifesciences.org).

**Sample-size estimation**

- You should state whether an appropriate sample size was computed when the study was being designed
- You should state the statistical method of sample size computation and any required assumptions
- If no explicit power analysis was used, you should describe how you decided what sample (replicate) size (number) to use

Please outline where this information can be found within the submission (e.g., sections or figure legends), or explain why this information doesn’t apply to your submission:

Sample size is reported together with every statistical comparison (and sample size summarized per experimental section is provided in supplementary table 1). While we are aware that formal power analysis should be done before experiments are conducted, this was not yet routinely adopted at the time we planned our study. Instead we estimated sample size based on results obtained in earlier works from our and other labs (e.g. Goltstein et al., J Neurosci, 2013).

**Replicates**

- You should report how often each experiment was performed
- You should include a definition of biological versus technical replication
- The data obtained should be provided and sufficient information should be provided to indicate the number of independent biological and/or technical replicates
- If you encountered any outliers, you should describe how these were handled
- Criteria for exclusion/inclusion of data should be clearly stated
- High-throughput sequence data should be uploaded before submission, with a private link for reviewers provided (these are available from both GEO and ArrayExpress)

Please outline where this information can be found within the submission (e.g., sections or figure legends), or explain why this information doesn’t apply to your submission:

In the manuscript, there is only one occasion of data (from a single animal) being excluded. The reason for this is explained in detail in the Methods (in brief, this regards data obtained using intrinsic signal imaging having blood vessel artifacts on which the automatic analysis was not able run).

**Statistical reporting**

- Statistical analysis methods should be described and justified
- Raw data should be presented in figures whenever informative to do so (typically when N per group is less than 10)
- For each experiment, you should identify the statistical tests used, exact values of N, definitions of center, methods of multiple test correction, and dispersion and precision measures (e.g., mean, median, SD, SEM, confidence intervals; and, for the major substantive results, a measure of effect size (e.g., Pearson's r, Cohen's d)
- Report exact p-values wherever possible alongside the summary statistics and 95% confidence intervals. These should be reported for all key questions and not only when the p-value is less than 0.05.

Please outline where this information can be found within the submission (e.g., sections or figure legends), or explain why this information doesn’t apply to your submission:

The full statistical reporting is done in the main text, always directly following the sentence in which the result is first mentioned, and includes test name, test statistics, exact p-value (when possible) and n. In order to maintain within reasonable length, figure legends state only statistical threshold and test method. We have strived to perform the statistical methods in -as systematic as possible- ways, and described these in detail in the Methods under the section statistical testing.

(For large datasets, or papers with a very large number of statistical tests, you may upload a single table file with tests, Ns, etc., with reference to sections in the manuscript.)

**Group allocation**

- Indicate how samples were allocated into experimental groups (in the case of clinical studies, please specify allocation to treatment method); if randomization was used, please also state if restricted randomization was applied
- Indicate if masking was used during group allocation, data collection and/or data analysis

Please outline where this information can be found within the submission (e.g., sections or figure legends), or explain why this information doesn’t apply to your submission:

Samples were allocated into groups for certain analyses, which was done using data from baseline measurements/responses, thus independent of the test comparisons. The group allocation method is described in the Main text, Methods and visualized in figure 3B and Figure 3-figure supplement 1.

**Additional data files (“source data”)**

- We encourage you to upload relevant additional data files, such as numerical data that are represented as a graph in a figure, or as a summary table
- Where provided, these should be in the most useful format, and they can be uploaded as “Source data” files linked to a main figure or table
- Include model definition files including the full list of parameters used
- Include code used for data analysis (e.g., R, MatLab)
- Avoid stating that data files are “available upon request”

Please indicate the figures or tables for which source data files have been provided:

We have made the experimental data available using a public repository: https://web.gin.g-node.org/pgoltstein/goltstein_meijer_pennartz_2018
